# Knowledge, attitude, and practices of patients and caregivers attending a Northern Nigerian family medicine clinic regarding the use of face mask during COVID-19 pandemic: a hospital-based cross-sectional study

**DOI:** 10.11604/pamj.2022.41.60.31253

**Published:** 2022-01-21

**Authors:** Abdulgafar Lekan Olawumi, Bukar Alhaji Grema, Abdullahi Kabir Suleiman, Godpower Chinedu Michael, Zainab Abdulazeez Umar, Abubakar Abiso Mohammed

**Affiliations:** 1 Department of Family Medicine, Aminu Kano Teaching Hospital, Kano, Nigeria,; 2 University of Maiduguri Teaching Hospital, Maiduguri, Nigeria

**Keywords:** Knowledge, attitude, practices, facemask, COVID-19, patients, caregiver

## Abstract

**Introduction:**

facemask use is well recognized as an effective public health strategy for preventing COVID-19. However, facemask can only provide enough protection if people recognize its importance and learn how to use it properly. The objective of the study was to assess the knowledge, attitudes, and practices (KAP) of patients or caregivers regarding the use of facemasks as a COVID-19 preventive measure and identifies the factors associated with its practice.

**Methods:**

a cross-sectional study where 480 patients or caregivers attending the Family Medicine Clinic were systematically selected over four weeks. A self-administered questionnaire was used to collect data on KAP regarding facemasks use. Student t-test and analysis of variance (ANOVA) were used to examine the relationship between the socio-demographic characteristics and KAP. Pearson's correlation coefficient was used to determine the relationship between knowledge, attitudes and practices. A p-value ≤ 0.05 was considered statistically significant.

**Results:**

about 82% of the respondents knew the correct steps of wearing a facemask, but with low positive attitudes. Further analyses showed that respondents were more likely to wear a facemask at clinics and public places than at home. There was a moderately strong correlation between knowledge and practices but weak correlations between attitude and knowledge, and attitude and practices of facemask use.

**Conclusion:**

the study revealed good knowledge and practices but low attitudes towards facemask use. Therefore, public health programmes or interventions on facemask usage as a COVID-19 preventive measure, should address the attitudinal problems and also involve the family and community leaders to enhance compliance.

## Introduction

Coronavirus disease 2019 (COVID-19) is a respiratory disease caused by severe acute respiratory syndrome coronavirus 2 (SARS-CoV-2), which first emerged in Wuhan city of China in December 2019, and since then has spread to over 200 countries around the world, resulting in a global pandemic [1,2]. The spread of the virus was mainly through respiratory droplets; which are released during coughing or sneezing and by touching contaminated surfaces or objects and then touching ones mouth, nose, or eyes [2]. It has an incubation period of approximately 1-14 days [2,3]. The first case of COVID-19 in Nigeria was confirmed on 27^th^ February 2020 involving an Italian businessman [4]. The number of cases steadily increased globally and as at 2^nd^ January, 2022, Nigeria; which is the most populous country in Africa has recorded more than 242,877 positive cases involving 3,033 deaths [2]. Kano state had a significant number of these cases and mortality due to denial, and misinformation leading to delay in adopting preventive measures [5,6].

The use of a face mask is one of the effective infection control measures recommended by the World Health Organization (WHO), and many countries including Nigeria adopted its use as part of their pandemic plans [7,8]. “A facemask is a loose-fitting and single-use device that covers the nose, mouth and chin” [9]. It is a simple and non-pharmaceutical individual intervention which provides a physical barrier against potentially infectious droplets [10]. The Centre for Disease Control (CDC) recommends the proper use of facemask to achieve the desired effect [9,10]. The proper use of facemask comprises the correct wearing technique and practice [10]. The proper technique of wearing facemask involves six steps: a) perform hand hygiene before putting on the facemask; b) ensure the mask covers the nose, mouth and chin, while tightening it against the sides of the face; c) perform hand hygiene before taking off the facemask; d) touch only the elastic bands during removal; e) ensure proper disposal of the used facemask in a paper bag or a lidded dust bin, f) perform hand hygiene after disposing of the facemask [11]. The correct practice of wearing facemask entails wearing it to protect yourself or others [10,11]. It is recommended for individuals to wear facemask to protect themselves when taking care of sick family members with fever or respiratory symptoms, visiting clinics or hospital, interacting with people who are not your household members and in the public gathering [11]. Individuals with fever or respiratory symptoms and those diagnosed with COVID-19 infection, even without symptoms are required to protect others by using facemask [7,11]. Resource-poor settings like Nigeria; where the incidence of infectious disease is high coupled with poor nutritional and environmental conditions, will rely heavily on a facemask to protect its citizens against COVID-19 and to prevent cross-contamination in the community [12]. That was why the Nigeria Centre for Disease and Control (NCDC) strongly recommends the use of facemask in the community and also emphasised its correct use [8].

Despite the importance of community facemasks usage in mitigating the spread of COVID-19 pandemic, and the strong recommendation by the WHO, CDC, and NCDC, there is no population-based study as at the time of this write-up in Nigeria that assesses the awareness, attitude and practice or compliance of people to this recommendation of wearing facemask. A study conducted by Ho HSW at a clinic in Hong Kong on knowledge, attitude and awareness on facemask usage revealed that 52% of the respondents knew the correct steps in wearing facemask with a positive attitude [13]. He also reported that a high percentage of outpatients and their caregivers are likely to wear facemask when visiting clinics to protect others (91.8%) and protect oneself (81.9%) [13]. Similarly, an online survey in Malaysia reported that 51.2% of the respondents wear facemask correctly [14]. A community-based study by Sikakulya *et al*. in Uganda reported 60.1% satisfactory knowledge on the use of face masks which favours respondents with tertiary level of education [15]. Also, 69.4% of the respondents were confident enough to correctly put on a face mask; 83.4% believed that a face mask can protect against COVID-19 and 75.9% of respondents had never shared their face mask [15]. The majority of respondents (95.2%) agreed wearing face masks in public places was important to protect them against COVID-19; 60.3% reported washing their hands before wearing and after removing the face mask. However, 51.5% reported removing the face mask if they needed to talk to someone [15]. However, an online survey in north-central Nigeria, by Reuben *et al*. reported positive attitudes toward the adherence to preventive measures against COVID-19 such that 82.3% of the respondents were using facemask [12]. The differences in findings amongst these studies may be due to the discrepancy between population-based and online-based studies. It could also be due to the change in knowledge and attitudes over time, coupled with differences in the socio-economic and cultural values amongst countries. The purpose of this study is to assess the knowledge, attitude, and practice of outpatients and caregivers on the use of facemask as a preventive measure against COVID-19 as well as identify associated factors that determine the practice in order to effectively control and mitigate the spread of the virus, and to advocate to the health authorities on the development and implementation of behavioural change programs to improve its use. This could also provide data for further studies. The alternate hypothesis for this study states that there is no correlation between knowledge, attitude and practices of facemask use among the participants.

## Methods

**Study design and setting:** this was a descriptive cross-sectional study conducted in the Family Medicine Clinic (FMC) of a tertiary hospital in Kano. Kano is the largest commercial center in Northern Nigeria, and it attracts a population with varied religions, ethnicity and occupation [16]. The hospital has 20 departments with about 800 beds´ capacity and serves as a referral center to the neighboring states and countries. The FMC is the primary care unit of the hospital, where patients are assessed, treated and referred to other units of the hospital.

**Study population and eligibility criteria:** the study population comprised adult (≥ 18 years) male and female patients or caregivers who presented at the clinic over four weeks. Consenting adult patients attending the clinic during the study period were recruited while those who need emergency care or with major neuropsychiatric illnesses such as schizophrenia were excluded from the study because they might not cooperate with the research processes.

**Sample size estimation:** considering 71.3% response rate of previous similar study [13] and after finite correction, a sample size of 506 was estimated using the formula [17]:


$$n=\frac{Z\alpha^{2}pq}{d^{2}}$$

where; n = minimum sample size, Z_α_= standard normal deviate corresponding to a 5% level of significance (1.96), P = (52% of the respondents in a similar setting in Hong Kong knew the correct steps in a wearing facemask with positive attitude) [13]. q = 1-p (48%), complementary probability of wearing facemask. d = level of precision which was set as 5%. The hospital record revealed an average of 250 adult patients seen daily in the FMC with about 20% (50) of them accompanied by a caregiver. This equals 300 adult individuals visiting the FMC daily. Therefore, the sampling frame will be 6,000 (300 x 5 x 4).

**Sampling method:** a systematic random sampling method was used to recruit 506 adult patients attending the FMC, within the sampling frame of 6,000 and sample interval of 12 (6,000/506). On the first day, the first respondent was chosen through balloting thereafter, every 12^th^ patient was recruited if he or she fulfilled the inclusion criteria.

**Study protocol:** after obtaining written informed consent, each participant was interviewed by the trained research assistant (residents and medical officers) using a pretested serially coded self-administered questionnaire. The questionnaire comprises two parts: 1) basic socio-demographic characteristics including age, gender, marital status, ethnicity, religion, educational level, occupation and brief family history; 2) knowledge, attitude and practice (KAP) which was adapted from a similar study on influenza-like illness (ILI) in Hong Kong [13]. The study also adapted it from the guidelines recommended by the Centre for Health Protection and the Centers for Disease Control and Prevention [9,11]. Some adjustments were made in the KAP questions to conform with the COVID-19 pandemics and the peculiarities of Nigerian socio-economic status as recommended by the Nigeria Centre for Disease Control (NCDC) [9]. Public health and epidemiological experts assessed the questionnaire on the relevance and correctness of the Knowledge, Attitude and Practice (KAP) questions regarding facemask usage, and appropriate adjustments were made. Questions concerning practices assessed the procedure of wearing a facemask, its use in public places, hospital/clinic and at home, and whether the facemask was used for self-protection or to protect others.

Respondents were asked 13 questions on the socio-demography, 9 on knowledge, 20 on attitude and 13 on the practice of wearing facemasks, to give a total of 55 questions. Regarding knowledge, a score of 1 was allotted to a correct answer and 0 for each incorrect or unsure answer. The final score ranged from 0 - 9. Attitude scores were calculated by giving -1 for a negative attitude, 0 for uncertain and 1 for a positive attitude favouring facemasks. The final score ranged from -20 to 20. The practice scores were calculated by scoring 0 for “no”, 1 for “sometimes”, and 2 for “yes”, to wearing a facemask. The final score ranged from 0 - 26.

**Data analysis:** data was stored in a pass-worded computer to ensure confidentiality and analysed using Statistical Package for Social Sciences version 23 (SPSS) statistical software. Qualitative data were described using frequency and percentages while quantitative data were described with mean and standard deviation. Student t-test and one-way analysis of variance (ANOVA) were used to examine the relationship between the socio-demographic characteristics and KAP. Pearson´s partial correlation coefficient was used to determine the relationship between knowledge, attitudes and practices. A p-value ≤ 0.05 was considered statistically significant.

**Ethical approval:** the ethical approval (No. NHREC/28/01/2020/AKTH/EC/2982) was obtained from the Research Ethical Committee of AKTH, Kano.

## Results

A total of 506 outpatients and caregivers were recruited for the study however, 480 completed the study to give a response rate of 95%. The respondents´ ages ranged from 18 to 86 years with a mean age of 33.34 (SD ±11.77) years. As shown in Table 1, most (74.4%) respondents fell within the 18-39 years age group and were predominantly females (57.9%). The majority were married (57.7%), Muslims (89.4%) with tertiary level of education (62.3%). A significant percentage of them were either self-employed (22.7%) or worked in public sectors (29.8%), with the majority (49.4%) earning below ₦30,000 (73USD) per month. The majority were non-smokers (94.4%), had never been diagnosed with COVID-19 infection (91.9%), and had no family members who had previously been diagnosed with the disease (85.0%).

**Table 1:** respondents’ socio-demographic characteristics

Variables	Frequency	Percentage
**Age (years)**		
18-39	357	74.4
40-59	108	22.5
60-79	14	2.9
≥ 80	1	0.2
**Sex**		
Male	202	42.1
Female	278	57.9
**Marital status**		
Single	186	38.8
Married	277	57.7
Divorced/separated	7	1.5
Widow	10	2.1
**Religion**		
Islam	429	89.4
Christianity	51	10.6
**Educational level**		
No formal	57	11.9
Primary	30	6.3
Secondary	94	19.6
Tertiary	299	62.3
**Occupation**		
Public sector	143	29.8
Private sector	59	12.3
Self employed	109	22.7
Unemployed	85	17.7
Retired	6	1.3
Schooling	74	15.4
Others	3	0.6
**Monthly income**		
(< 73USD)	237	49.4
(73-146USD)	165	34.4
(147-219USD)	27	5.6
(>219USD)	51	10.6
**Cigarette smoking**		
Yes	27	5.6
No	453	94.4
**Previously diagnosed with COVID-19**		
Yes	39	8.1
No	441	91.9
**Family member had COVID-19 before**		
Yes	72	15.0
No	408	85.0

**Knowledge on the use of facemask:** the overall knowledge of the respondents had a mean score of 6.47 (SD ±1.82), which is equivalent to 72% of the total score. As was shown in Table 2, the majority of the respondents (92.3%) knew the main clinical symptoms; modes of spread (81.3%) and the importance of facemask usage (87.5%) to limit the spread of COVID-19 infection. However, only 57.1% of them knew that there is no effective cure for COVID-19 infection and 39.4% of them recognized that a surgical facemask is more effective than a fabric facemask. A significantly lower percentage (25%) of the respondents knew that when wearing a face mask, it was still required to cover one's mouths while coughing and sneezing, and that a used facemask cannot be re-used, even if the wearer is not ill (43.3%). A higher percentage of the respondents (81.9%) knew how to wear a facemask correctly, and in the absence of commercial or surgical facemask; they can use home-made fabric facemask (78.1%).

**Table 2:** knowledge on the use of facemask to reduce the spread of COVID-19

Questions	Yes (%)	No (%)	I don't know (%)
The main clinical symptoms of COVID-19 are fever, fatigue, dry cough, and body aches	443 (92.3)	13 (2.7)	24 (5.0)
There is currently no effective cure for COVID-19	274 (57.1)	118 (24.6)	88 (18.3)
The COVID-19 virus spreads via respiratory droplets of infected individuals	390 (81.3)	30 (6.3)	60 (12.6)
Using facemask is important to tackle COVID-19	420 (87.5)	36 (7.5)	24 (5.0)
A cloth facemask is as effective as a regular surgical facemask	227 (47.3)	189 (39.4)	64 (13.3)
If I am not sick, the used medical face mask can be stored in a bag for later use	208 (43.3)	225 (46.9)	47 (9.8)
In the absence of commercial or surgical facemask the general public can use household made cloth facemask	375 (78.1)	66 (13.8)	39 (8.1)
When wearing a facemask at the clinic, there is no need to cover your mouth when sneezing or coughing	337 (70.2)	120 (25.0)	23 (4.8)
I know the correct procedure in wearing facemask	393 (81.9)	56 (11.7)	31 (6.5)

**Attitude towards the use of facemask:** the respondents' attitudes had a mean score of 7.49 (SD ± 5.68), which is equivalent to 37.5% of the total score. As shown in Table 3, 70.8% of respondents believed that they were more likely to contract COVID-19 at the clinic, and 64.6% thought the risk of contracting COVID-19 was higher in the clinics than in public places. Only 50% of respondents believe COVID-19 infection is still a concern, and 58.8% believe they are more susceptible to COVID-19 infection in the public places than clinics. The majority of respondents (75.4%) stated that contracting COVID-19 is serious and would be difficult since the infection can be passed on to family members (79.4%) and even result in job absence (73.3%). Most respondents (87.5%) believed that wearing a facemask is a good way to protect oneself against COVID-19 infection but only 39.4% stated that wearing a facemask cannot fully prevent the transmission of COVID-19 infection (Table 3).

**Table 3:** attitude towards the use of facemask to reduce the spread of COVID-19

Questions	Agree (%)	Disagree (%)	Uncertain (%)
Perceived susceptibility			
I am more susceptible to COVID-19 infection at the clinic than in public places.	340 (70.8)	92 (19.2)	48 (10.0)
There is a high chance of having COVID-19 infection transmitted to me while I am at the clinic.	310 (64.6)	128 (26.7)	42 (8.8)
I feel that since the cases of COVID-19 is reducing; I no longer have to worry about contracting it.	201 (41.9)	240 (50.0)	39 (8.1)
I feel that I am susceptible to getting COVID-19 infection in the public places.	282 (58.8)	142 (29.6)	56 (11.7)
Perceived severity			
I believe that getting COVID-19 infection is serious.	362 (75.4)	75 (15.6)	43 (9.0)
Having COVID-19 will be troublesome for me as I may spread it to loved ones.	381 (79.4)	69 (14.4)	30 (6.3)
Having COVID-19 will be troublesome for me as I have to take time off work.	352 (73.3)	96 (20.0)	32 (6.7)
Perceived benefit of wearing a facemask			
I believe that wearing a facemask is a good way to protect myself against the COVID-19 infection at the clinic.	420 (87.5)	33 (6.9)	27 (5.6)
At the clinic, wearing a facemask cannot fully prevent the transmission of the COVID-19 infection.	189 (39.4)	254 (52.9)	37 (7.7)
Cues to action			
I would wear a facemask if there were more posters to remind me.	270 (56.3)	175 (36.5)	35 (7.3)
If the doctor or nurse tells me to, I will wear a facemask.	351 (73.1)	98 (20.4)	31 (6.5)
I will wear a facemask without anybody telling me.	393 (81.9)	58 (12.1)	29 (6.0)
Self-efficacy			
I know the proper steps for putting on a facemask.	414 (86.3)	38 (7.9)	28 (5.8)
I know the proper steps for putting off a facemask.	409 (85.2)	40 (8.3)	31 (6.5)

**Figure 1:**
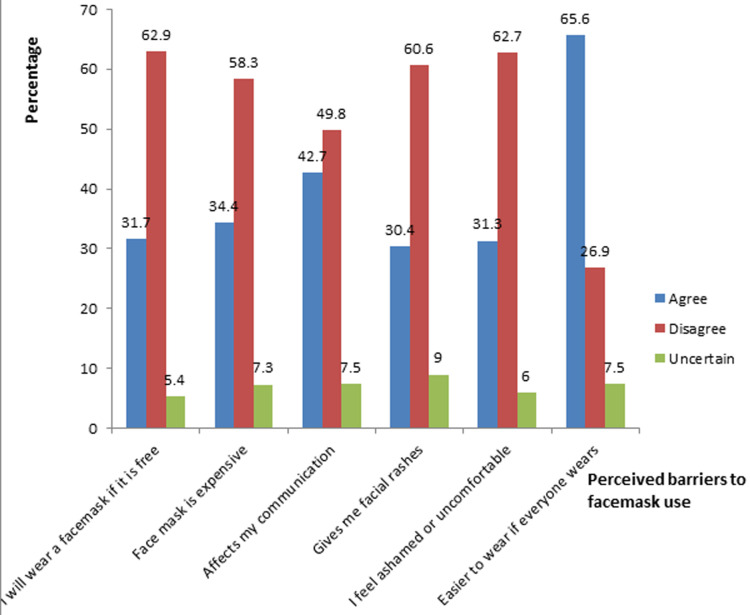
perceived barriers to facemask use

The majority of respondents (81.9%) indicated that they would use a facemask if no one informed them, and 73.1% would be more likely to wear one if a nurse or doctor reminded them. However, only 56.3 % opined that seeing posters would make them more likely to use a facemask. Most of respondents (86.3%) believed that they knew the proper procedure for wearing and removing (85.2%) a face mask (Table 3). Figure 1 depicted the identified perceived barriers to wearing a mask, with 62.9% disagreeing with the statement, “I would only use a face mask if it was free.” and 58.3% of the respondents disagreed that facemask is expensive. Furthermore, 42.7% responded that wearing a facemask made it difficult to communicate, and 30.4% stated that wearing facemask caused facial rashes. A substantial number of respondents (62.7%) disagreed that they would feel ashamed or uncomfortable if they were the only one wearing a facemask, but 65.6% agreed that if everyone else was wearing a facemask, it would be easier for them to wear one as well.

**Practices involving the use of facemask:** the respondents' practices had a mean score of 21.23 (SD ± 4.20), which is equivalent to 81.7% of the total score. Practices were divided into four categories, which include the general facemask usage, home, public and clinic practices. These practices were either to protect oneself against COVID-19 or to protect others against COVID-19. The majority of the respondents had good general facemask usage practices which involved; performing hand hygiene before wearing facemask (67.9%), tightening the facemask against the side of the face (62.7%), covering the nose, mouth and chin with facemask (85.4%), fitting the metal strip on the nose (65.8%), touching only the elastic band during removal (75.6%), proper disposal after use (73.3%), and washing fabric facemasks for re-use (67.1%). Respondents were more likely to report wearing a face mask to protect themselves in public places (81.9%) and at the clinic (81.3%) than at home (59.8%) when taking care of sick family members. The use of face masks at home (59.0%) was also lower than that in public places (78.5%) and at the clinic (85.4%) for protecting others (Table 4).

**Table 4:** practices involving the use of facemask to reduce the spread of COVID-19

Questions	Yes (%)	Sometimes (%)	No (%)
I used to perform hand hygiene before putting on the facemask.	326(67.9)	132 (27.5)	22 (4.6)
I used to tighten the facemask against the sides of my face.	301 (62.7)	109 (22.7)	70 (14.6)
My facemask used to cover my nose, mouth and chin.	410 (85.4)	50 (10.4)	20 (4.2)
I used to fit the metal strip on my medical facemask on my nose.	316 (65.8)	91 (19.0)	73 (15.2)
I only touch the elastic band during removal of my facemask.	363 (75.6)	84 (17.5)	33 (6.9)
I used to drop the facemask in a rubbish bag or dustbin after use.	352 (73.3)	83 (17.3)	45 (9.4)
I re-used my cloth facemask after washing.	322 (67.1)	88 (18.3)	70 (14.6)
At Home			
I wear facemask at home when caring for family members with fever or respiratory symptoms and when hosting visitors.	287 (59.8)	107 (22.3)	86 (17.9)
I wear facemask at home when I have fever or respiratory symptoms.	283 (59.0)	110 (22.9)	87 (18.1)
In Public			
I wear facemask when going to public places and when boarding public vehicles.	393 (81.9)	72 (15.0)	15 (3.1)
I wear facemask when I have fever or respiratory symptoms in public places or boarding public vehicles.	377 (78.5)	77 (16.0)	26 (5.4)
In the Hospital/Clinic			
I wear facemask when taking a sick family member to the hospital or clinic.	390 (81.3)	76 (15.8)	14 (2.9)
I wear facemask when going to hospital or clinic for the treatment of my ailment.	410 (85.4)	64 (13.3)	6 (1.3)

**Association between socio-demographic characteristic and knowledge, attitude and practices of facemasks use:** there was no statistically significant association between the knowledge of the respondents and their socio-demographic characteristics (Table 5). There was significant association between respondents´ attitude and their marital status (F = 3.732, p = 0.011), monthly income (F = 2.938, p = 0.033) and those with previous history of COVID-19 infection in their family members (t = -2.046, p = 0.041). The widowed (11.80) and respondents with monthly income (8.26) below ₦30,000 (73USD) got the highest and most significant mean score for attitude toward the usage of a facemask (Table 5). Furthermore, there was significant association between the facemask practices and occupation (F = 2.190, p = 0.043) of the respondents, such the unemployed (22.15%) and those working in public sectors (21.69) had the best facemask practices on average (Table 5).

**Table 5:** association between socio-demographic characteristic and knowledge, attitude and practice of facemask use

Variables	Knowledge			Attitude			Practice		
	Mean(SD)	F	p-value	Mean(SD)	F	P–value	Mean(SD)	F	P -value
									
Age (years)		1.176	0.318		0.479	0.697		0.479	0.697
18-39	6.55(1.79)			7.67(5.65)			21.34(4.01)		
40-59	6.22(1.86)			6.94(5.72)			20.80(4.81)		
60-79	6.29(2.09)		7.21(6.39)				21.93(3.89)		
≥ 80	5.00			8.00			19.00		
Sex		0.240**	0.810		-0.370**	0.711		-0.370**	0.711
Male	6.49(1.63)			7.38(5.43)			21.10(4.27)		
Female	6.45(1.95)			7.58(5.85)			21.32(4.14)		
Marital status		1.225	0.300		3.732	0.011*		3.732	0.011*
Single	6.53(1.64)			8.13(5.42)			21.20(3.91)		
Married	6.46(1.87)			6.92(5.73)			21.32(4.39)		
Divorced/ separated	6.57(3.05)			7.00 (6.30)			19.71(4.72)		
Widow	5.40(2.37)			11.80(5.94)			20.20(3.82)		
Religion		0.880**	0.380		0.747**	0.636		0.747**	0.636
Islam	6.49(1.77)			7.54(5.64)			21.35(4.21)		
Christianity	6.25(2.20)			7.14(6.01)			20.22(3.97)		
Educational level		1.023	0.395		2.245	0.063		2.245	0.063
No formal	6.36(2.07)			8.84(5.69)			22.72(2.99)		
Primary	6.90(1.95)			9.63(6.34)			21.13(3.88)		
Secondary	6.22(2.19)			7.44(5.62)			21.05(4.56)		
Tertiary	6.53(1.60)			7.06(5.52)			21.02(4.23)		
Occupation		1.214	0.298		1.378	0.222		1.378	0.222
Public sector	6.64(1.76)			7.27(6.07)			21.69(4.16)		
Private sector	6.00(2.22)			8.97(6.26)			20.76(4.20)		
Self employed	6.53(1.80)			7.70(5.45)			20.84(4.51)		
Unemployed	6.49(1.94)			7.45(5.55)			22.15(3.94)		
Retired	6.33 (0.52)			9.17(4.17)			20.00(4.42)		
Schooling	6.46 (1.46)			6.36(4.85)			20.47(3.87)		
Others	5.00(2.00)			9.67(2.08)			17.33(3.51)		
Monthly income		0.094	0.963		2.938	0.033*		2.938	0.033*
(< 73USD)	6.48(1.77)			8.26(5.32)			21.22(4.31)		
(73-146USD)	6.50(1.88)			6.73(6.09)			21.15(4.25)		
(147-219USD)	6.33(2.17)			6.44(6.27)			21.85(3.10)		
(>219USD)	6.39(1.70)			6.94(5.21)			21.22(4.02)		
Cigarette smoking		-1.468**	0.153		1.210**	0.236		1.210**	0.236
Yes	5.78(2.55)			9.07(7.06)			21.59(3.82)		
No	6.51(1.76)			7.40(5.58)			21.21(4.22)		
Previously diagnosed with COVID-19		0.624**	0.533		0.257**	0.797		0.257**	0.797
Yes	6.64(1.72)			7.72(6.31)			22.00(3.31)		
No	6.45(1.83)			7.47(5.62)			21.16(4.26)		
Family member had COVID-19 before		-1.168**	0.244		-2.046**	0.041*		-2.046**	0.041*
Yes	6.24(1.64)			6.24(6.11)			21.33(3.46)		
No	6.51(1.85)			7.72(5.57)			21.21(4.31)		

* Significant, SD: standard deviation, df: degree of freedom, **t-test

**Correlation between knowledge, attitude and practice:** knowledge and attitude (r = 0.272, p 0.001), as well as attitude and practices (r = 0.126, p = 0.006) towards facemask usage, had a positive but weak correaltion. Knowledge and practices also had a positive but moderately strong correlation (r = 0.314, p 0.001) Table 6.

**Table 6:** correlation coefficient analysis between knowledge, attitude and practice

Variables	Correlation coefficient (r)	p-value
Knowledge and attitude	0.272	< 0.001*
Knowledge and practice	0.314	< 0.001*
Attitude and practice	0.126	0.006*

Level of significance at < 0.01

## Discussion

Globally, facemasks have been accepted as one of the most effective public health interventions for reducing the spread of COVID-19 infection. Face masks, on the other hand, can only give good protection if the public understands the importance and knows how to use them properly [7]. In this study, the respondents demonstrated a high knowledge (72%), good practices (81.7%) but low positive attitude (37.5%) towards the use of facemasks for the prevention of COVID-19 infection. This was also corroborated with the moderately strong correlation between knowledge and practices compared to the weak correlations between attitude and knowledge, and attitude and practices of facemask use. Regardless, the respondents´ attitude still had a positive but weak correlation with their knowledge and practices of facemask use.

The high knowledge on facemask usage in this study was similar to the findings of other studies [13,15,18,19]. This finding was contrary to that of Kumar *et al*. in Pakistan who reported an unsatisfactory knowledge of facemask usage among healthcare workers [20]. Apart from the difference in population, the study was conducted during the peak of COVID-19 infection with lots of controversies and confusing messages on social media [20]. Although, there was no significant relationship between respondents knowledge and their socio-demographic characteristics, majority of the respondents knew the main clinical symptoms, modes of spread and the importance of facemask usage to limit the spread of COVID-19 infection. However, a lower proportion (25%) of the respondents knew that when wearing a face mask, it was still required to cover one's mouth while coughing and sneezing, and that a used facemask cannot be re-used, even if the wearer is not ill (43.3%). This finding was similar to that reported by Ho HSW in Hong Kong [13]. This implies that respondents were unaware that facemasks could only act as a barrier for large droplets and not extremely minute particle aerosols, which are released during sneezing [21]. This misunderstanding could lead to an increase in the spread of various respiratory tract infections that can be transmitted through the facemask. These findings point to the urgent need for a better public health campaign to raise awareness about the proper use of facemasks.

The level of positive attitudes towards the use of facemask to limit the transmission of COVID-19 was low compared to other studies [13,15,18,19]. This could likely be related to the cultural misconceptions and misinformation in Nigeria that led to initial denial and delayed adoption of preventive measures against COVID-19 infection [5,22]. This low positive attitude was also reported towards Lassa fever infection, prevention and control by Ukwenya *et al*. in Owo, south-western Nigeria [23] and towards the use of personal protective equipment (PPE) for Prevention of COVID-19 by healthcare workers in Ogbomoso, south-western Nigeria by Alao *et al*. [24]. A low positive attitude towards the use of facemask was also reported among healthcare workers by Kumar *et al*. in Pakistan probably due to earlier stated reasons [20]. Nonetheless, majority of the respondents believed that contracting COVID-19 is a serious infection and that wearing a facemask is a good way to protect oneself against being infected. Interestingly, only 56.3 % opined that seeing posters would make them more likely to use a facemask. Similar findings was reported in a similar outpatient setting in Hong Kong [13]. This was attributable to the fact that there are numerous health reminder posters in the waiting area and majority of the instructions on these posters were written, which may be a problem for those who have difficulty reading.

This study also reported a significant association between attitude and marital status and income, which was similar to the findings of Sikakulya *et al*. in Uganda [15]. Respondents who were poor (< 73USD monthly income), single or widowed had high positive attitude towards facemask usage. This could be because these are the vulnerable groups in the community and they are likely to experience the worse consequences of COVID-19 infection hence, they adhere to the precautionary measures. Furthermore, respondents with previous history of COVID-19 infection in their family members were also noticed to have a significant and better attitude towards the use of facemask (t = -2.046, p = 0.041). Although, there was no study to compare with, respondents who have had a family member previously diagnosed with COVID-19 infection will not want to go through the same ordeal again, so they will take all necessary precautions. Another important findings in this study was that majority of the respondents (65.6%) agreed that they would be encouraged to wear facemask if others also wear. Policy makers could use this as reason to enforce facemask usage on people so as to enhance wide acceptance and usage.

Similar to previous studies, this study also revealed a significant practice of good facemask use among the respondents [13,15,18,19]. Majority of the respondents had good general practices of facemask use however; they are more likely to wear facemask to protect themselves or others in the public places and clinics than at home. This was attributed to the feeling of being safe at home hence; this highlights the importance of ensuring correct knowledge and understanding of wearing a facemask. Furthermore, there was significant association between the facemask practices and occupation (F = 2.190, p = 0.043) of the respondents, such that the unemployed and those working in public sectors had the best facemask practices on average. This could be because wearing a facemask in most public places in Kano, and even Nigeria as a whole, is compulsory by government policy; hence, civil officials and job seekers must conform before entering any government facilities [25]. Although the majority of the respondents in this study had adequate knowledge and were aware of the benefits of wearing a facemask as well as the risks of not wearing one, government regulation was just a contributing factor to its use.

On the contrary, a community-based study by Lee *et al*. in Hong Kong reported an unsatisfactory practices and techniques of using facemask to prevent COVID-19 infection [10]. This could be due to the differences in the settings and tools used in each study.

**Strengths and limitations of the study:** to prevent non-response bias, the questionnaire was tranlated into the major local language (Hausa) by a professional translator to accommodate the illiterates who may have been unwilling to participate. The option of having an interviewer to read the questions to them was also offered to those who could not read at all. Also, a probability sampling method was used to ensure that the consenting patients and caregivers had an equal chances of being selected for the study. To the best of our knowlegde, this is probably one of the first population-based study assessing the use of facemask to the prevent COVID-19 infection in Nigeria. This study had some limitations. The use of closed-ended questions may not have covered the whole range of answers relating to facemask use, thus a qualitative or informal interview may be required in further studies. Also, the genralisation of the results of a hospital-based descriptive cross-sectional study of this type should be done with caution. Despite all these limitations, this study still provides valuable insights for further investigation on the knowledge gaps in the correct use of face masks. Future studies should include a community-based study to explore other factors related to the knowledge, attitude and practices on facemask use.

## Conclusion

This study demonstrated adequate knowledge and practices but low positive attitude towards facemask use. It also revealed a weak positive correlation between attitude and the other two parameters. Marrital status, income, previous history of COVID-19 infection in a family member and occupation were the significant factors associated with the attitude and practices of facemask usage respectively. Therefore, interventions or public health programmes on facemask usage as a preventive measure for COVID-19 infection, should address the attitudinal problems and also involve the family and community leaders so as to foster community ownershipship and participation.

**Funding:** the study was fully funded by the researchers.

### What is known about this topic

- *Facemask use is well recognised as an effective public health strategy for preventing COVID-19 infection;*
- *Most studies on the knowledge, attitude and practice of facemask use are online surveys*.

### What this study adds

- *To the best of our knowledge, this is probably one of the first population-based study assessing the use of facemask to the prevent COVID-19 infection in Nigeria;*
- *Determine the factors associated with the knowledge, attitude and practice of facemask use*.
